# Valosin-containing protein, neural proteopathies, and implications for neural regeneration

**DOI:** 10.4103/NRR.NRR-D-25-00442

**Published:** 2025-07-05

**Authors:** Jae-Geun Lee, Eun-Ji Lee, Hoon Ryu, Jeong-Soo Lee

**Affiliations:** Microbiome Convergence Research Center, Korea Research Institute of Bioscience and Biotechnology (KRIBB), Daejeon, Republic of Korea; KRIBB School, University of Science and Technology, Daejeon, Republic of Korea; Center for Brain Disorders, Brain Science Institute, Korea Institute of Science and Technology (KIST), Seoul, Republic of Korea; KHU-KIST Department of Converging Science and Technology, Kyung Hee University, Seoul, Republic of Korea

Proteostasis, also known as protein homeostasis, is a tightly regulated cellular quality control process that ensures the balance of protein synthesis, folding, posttranslational modifications, and degradation. Maintaining proteostasis is vital for cellular function, organismal health, and longevity. The disruption of proteostasis can lead to a range of detrimental effects, including accelerated aging, compromised cellular function, and even cell death, manifesting in numerous human diseases (Hipp et al., 2019). Neurodegenerative diseases, such as Alzheimer’s, Parkinson’s, and Huntington’s diseases (AD, PD, HD, respectively), are often characterized by the accumulation of misfolded proteins that aggregate into granules or inclusions. These aggregates form when proteins lose their proper conformation and fail to be refolded or degraded efficiently due to the failure of proteostasis. The persistence of these pathological protein aggregates can interfere with cellular processes, disrupt organelle function, and ultimately contribute to disease progression (Hipp et al., 2019).

Cells rely on a sophisticated network of molecular chaperones and their co-chaperones to maintain proteostasis. Molecular chaperones are specialized proteins that assist in the correct folding of newly synthesized polypeptides, preventing misfolding and aggregation, and help refold partially misfolded proteins, restoring their functional state, while co-chaperones modulate the specificity and the binding/release cycle with target substrates (Gu et al., 2025). In cases where proteins are irreversibly damaged, chaperones direct them toward degradation pathways, such as the ubiquitin-proteasome system (UPS) or autophagy-lysosomal pathway (ALP), to prevent the accumulation of toxic aggregates (Chu et al., 2023). The regulation of proteostasis becomes increasingly challenging with age, as the efficiency of chaperone systems and degradation pathways declines, which contributes to the accumulation of damaged and misfolded proteins and increases the risk of age-associated diseases, particularly neurodegenerative disorders (Hipp et al., 2019).

Recent studies, including our own, have reported that valosin-containing protein (VCP)/p97 (also known as Cdc48 in yeast and plants, CDC-48 in worms, and Ter94 in flies), one of the key molecular chaperones, is involved in regulating neurodegenerative diseases through various mechanisms (Darwich et al., 2020; Giong et al., 2024). Here, through the perspective of these studies, we will discuss the important and novel role of VCP and its therapeutic implications for neurodegenerative diseases, and, potentially, neural regeneration.

**Diverse roles of valosin-containing protein in proteostasis:** VCP is a type II AAA^+^ (ATPases Associated with diverse cellular Activities) ATPase and a molecular chaperone, essential for cellular homeostasis and protein quality control. It functions within a complex network of cofactors such as p47 and Ufd1/Npl4 to extract ubiquitinated proteins for recycling or degradation. Structurally, VCP forms a hexamer with an N-terminal domain for substrate selection, two ATPase domains (D1, D2), and a C-terminal tail for cofactor interactions. Its ATPase activity, primarily driven by the D2 domain, enables substrate unfolding and degradation, while ATP binding to D1 promotes hexamer formation and ATP hydrolysis in D2 induces conformational changes necessary for its chaperone function. VCP shuttles between the nucleus and cytoplasm via its nuclear localization sequence and C-terminal phosphorylation (pS-612, pT-613), with cofactors p37 and VCF1/2 regulating VCP import and nuclear accumulation (Chu et al., 2023; Wrobel et al., 2024). Although no nuclear export signal has been identified, palmitoylation may assist in membrane association. Client proteins are processed through the central or lateral openings of the hexamer, with cofactor interactions and posttranslational modifications modulating the ATPase activity (Chu et al., 2023).

VCP facilitates the degradation of misfolded proteins via the UPS and ALP to ensure proteostasis, collaborating with CHIP/STUB1 and Bag-1. VCP not only promotes selective autophagy such as mitophagy, lysophagy, pexophagy, and nucleophagy but also plays a pivotal role in endoplasmic-reticulum-associated degradation followed by UPS, with the interactions of cofactors p47 and ATL1, to maintain endoplasmic-reticulum homeostasis and regulate dendritic spine formation. For the ALP, VCP has been implicated in the autophagy induction by regulating the Beclin-1 protein level and the phosphatidylinositol-3-kinase complex, and potentially in the fusion of the autophagosome and the lysosome because VCP knockdown or expression of VCP with genetic mutations resulted in the autophagosome accumulation and the failure of the aggregated protein. In addition to these functions, VCP also modulates mitochondrial homeostasis, membrane fusion, calcium uptake, and apoptosis (Chu et al., 2023). During ALP, VCP has been implicated in autophagy induction by regulating the assembly of phosphatidylinositol-3-kinase complex, as well as in autophagosome-lysosome fusion. Consistent with this function, VCP knockdown or expression of VCP with genetic mutations leads to autophagosome accumulation and failure to degrade aggregated proteins (Chu et al., 2023).

VCP modulators such as CB-5339 and KUS121 are currently being evaluated in clinical trials for hematologic malignancies (NCT04402541) and retinal ischemic disease (NCT06178055), respectively. Clinical trials for neurodegenerative diseases have not yet been initiated; however, based on the critical function of VCP in these diseases, VCP modulators may be considered as a potential new drug for their treatment (see below).

**Role of valosin-containing protein in neurodegenerative diseases:** Clinically, the expression of VCP is reduced in patients with age-related diseases. VCP colocalizes with Tau aggregates and its lower expression levels correlate with increased Tau protein and its phosphorylated forms in the cortices of AD patients (Giong et al., 2024). A mutant form of VCP (*VCP*^*D395G*^) that fails to disaggregate Tau resulted in pathological Tau aggregation and autosomal-dominant dementia, suggesting that VCP dysfunction exacerbates Tau pathology and neurodegeneration in AD (Darwich et al., 2020). Considering the multifaceted roles of VCP in the UPS and ALP for proteostasis that are specialized to remove protein aggregates, it was highly plausible that VCP promoted the clearance of accumulated Tau via the UPS or ALP in tauopathy and AD.

In our recent study, we addressed this possibility by adopting a zebrafish model that could offer a variety of advantages such as genetic tractability, real-time *in vivo* imaging at the cellular level, and an amenable high-content drug screening for both mechanistic understandings and drug development for neurodegenerative diseases. We generated a zebrafish tauopathy model in which human Tau^P301L^ mutant protein was overexpressed in a neuron-specific manner and observed rapid degradation of the Tau protein despite its continued transcriptional expression. This degradation was found to be autophagy-dependent, evidenced by the substantial accumulation of Tau protein upon either genetic (*atg5* knockdown) or chemical (chloroquine, NH₄Cl) inhibition of the autophagy pathway. Through the whole-transcriptome analysis, we discovered that expression of *vcp* mRNA was significantly upregulated in neurons expressing Tau protein and, conversely, as Tau protein underwent autophagic degradation, *VCP* mRNA and protein levels decreased correspondingly. In line with these findings, VCP morpholino knockdown led to significant Tau protein accumulation, successfully rescued by expressing synthetic wild-type *VCP* mRNA. Notably, expression of *VCP*^*D395G*^ (a mutation linked to vacuolar tauopathy; (Darwich et al., 2020) rescued Tau clearance defects upon VCP knockdown, mimicking wild-type *VCP*. In contrast, *VCP*^*DKO*^ (a null mutant) or *VCP*^*A232E*^ and *VCP*^*R155H*^ (mutations associated with multisystem proteopathy 1/MSP1) failed to restore Tau clearance, highlighting the complex functional impacts of distinct *VCP* mutations (Giong et al., 2024).

The role of VCP in Tau clearance in an autophagy-dependent manner was further confirmed in a Tau-overexpressing mouse model and a mouse hippocampal cell line. Tau protein overexpressed in the mouse hippocampal neurons was cleared *in vivo* by co-expression of VCP, of which effect was negated by chloroquine treatment. Also, the treatment of SMER-28, an VCP agonist, mimicked the autophagic Tau clearance function of VCP in the cell line. Furthermore, the behavioral and memory abnormalities caused by Tau overexpression in the mouse model were rescued by VCP overexpression. Importantly, in the brains of human AD patients VCP expression was significantly lower than in age-matched normal-aged brains, revealing an inverse correlation with levels of phosphorylated Tau. Our findings demonstrated that the tight regulation of VCP expression plays an essential role in maintaining the homeostasis of Tau protein via autophagy *in vivo*. This suggests that the regulation of VCP expression or its activity could be a potential therapeutic target for tauopathy and AD with interventions aiming autophagy-modulating function of VCP (Giong et al., 2024).

Similar to tauopathy and AD, VCP also plays an essential role in other neurodegenerative diseases including PD, HD, and Amyotrophic lateral sclerosis. VCP suppresses the aggregation of α-synuclein and TDP-43 proteins and prevents their propagation between neurons, and when VCP function is impaired or mutated, protein aggregation is promoted (Zhu et al., 2022). Notably, UP109 treatment, a VCP activator, reduced TDP-43 aggregates, suggesting its potential as a therapeutic approach for TDP-43 proteinopathy (Chu et al., 2023) and a VCP modulator Gossypol promotes the formation of the VCP-LC3-mHTT (mutant huntingtin) complex, enhancing the autophagic degradation of mHTT (Chu et al., 2023).

The detailed molecular mechanisms by which VCP promotes Tau clearance via autophagy are still elusive; based on its known roles in other contexts, VCP may enhance the autophagy flux at the initiation process of the autophagy, shuttle Tau protein more efficiently to the autophagosome by bridging Tau and LC3, promote the membrane fusion of the autophagosome and the lysosome, and/or replace damaged lysosomes (Giong et al., 2024).

**Neural degeneration and regeneration studies using the zebrafish model:** Restoring functional neurons by regeneration to replace degenerating ones could be one of the most effective strategies for treating neurodegenerative diseases. It is now widely accepted that the interplay between neural regeneration and proteostasis that ensures the proper folding, trafficking, and degradation of proteins is critical for maintaining neuronal health and promoting recovery from neurodegenerative diseases. During neural regeneration, a balanced proteostasis network can support neuronal survival, differentiation, and synaptic remodeling by eliminating damaged proteins and reducing cellular stress, creating a favorable environment for neuronal repair and functional recovery. As one of the key pathways for proteostasis, autophagy has been strongly implicated in regeneration by regulating a series of regeneration processes including sensing injury, cell cycle re-entry, proliferation, differentiation, and remodeling (Moreno-Blas et al., 2025). Based on the core function in protein degradation and the maintenance of cellular homeostasis, it is plausible that VCP, the molecular chaperone essential for proteostasis and autophagy, contributes to not only mitigating neural degenerative phenotypes but also modulating neural regeneration.

Unlike mammals, zebrafish harbors remarkable regenerative abilities upon damages for a series of tissues including the nervous system (Moreno-Blas et al., 2025), allowing dissection of underlying molecular mechanisms and identification of a novel target that may help to facilitate the mammalian neural regeneration. For example, in an adult zebrafish model with neurotoxic Aβ_42_ injected into the brain, regenerative neurogenesis occurred through neuron–glia interactions via the serotonin/BDNF/NGFR axis (Bhattarai et al., 2020). In addition, a high-content drug screening adopting dopaminergic neuron-specific chemogenetic ablation system using the zebrafish transgenic lines yielded the RAAS (Renin-angiotensin system) as a repurposing target for developing neuroprotective and neural regeneration drugs (Kim et al., 2021). Furthermore, a single-cell level RNA-seq analysis of the zebrafish adult spinal cord injury model delineated the detailed molecular signature during regeneration, with the identification of an unconventional, transient injury-responsive neuronal population indispensable for functional recovery (Saraswathy et al., 2024). Thus, zebrafish may serve as a versatile model organism to gain comprehensive insight into the intersection of neural degenerative and regenerative processes with regard to proteostasis and VCP function.

**Figure 1:**
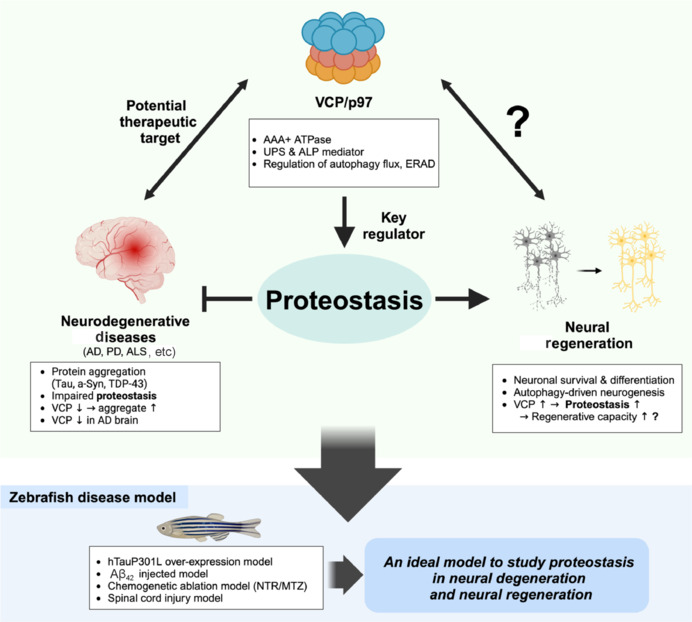
Role of VCP and proteostasis in neurodegeneration and neural regeneration using zebrafish. The balanced proteostasis is essential for preventing neurodegenerative diseases and promoting neural regeneration. As a central regulator of the cellular quality system for proteostasis, it is plausible that VCP contributes to not only mitigating neurodegenerative defects but also modulating neural regeneration by clearing harmful protein aggregates and maintaining proteostasis through the UPS and ALP. Elevating the reduced level of VCP in patients with age-related neurodegenerative diseases including AD and tauopathy could be a promising therapeutic strategy. This fascinating field of research can be benefited by utilizing the versatile zebrafish animal model that can offer valuable insights into both neural degeneration and regeneration. See details for texts. Created with BioRender.com. AAA+: ATPases Associated with diverse cellular Activities; AD: Alzheimer’s disease; ALP: autophagy–lysosome pathway; ALS: amyotrophic lateral sclerosis; a-Syn: alpha-synuclein; Aβ: amyloid-beta; ERAD: endoplasmic-reticulum-associated degradation; MTZ: metronidazole; NTR: nitroreductase; PD: Parkinson’s disease; TDP-43: TAR DNA-binding protein 43; UPS: ubiquitin-proteasome system; VCP: valosin-containing protein.

**Concluding remarks:** Recent studies have highlighted the essential roles of VCP in neurodegeneration, demonstrating its involvement in clearing pathological protein aggregates and maintaining proteostasis through the UPS and ALP. Simultaneously, such a balanced proteostasis network is also critical to support neuronal survival, differentiation, and functional recovery during neural regeneration. VCP, as a central regulator of the cellular quality system and autophagy, is also highly likely to be involved in these regenerative processes by facilitating the degradation of damaged proteins and monitoring neuronal health. With the help of zebrafish models that allow an array of genetic manipulations and real-time *in vivo* imaging, novel perspectives on the interplay between proteostasis and neural degeneration/regeneration, mediated by molecular chaperones such as VCP, may eventually allow us to devise an unprecedented therapeutic avenue for curing neurodegenerative diseases (**Figure 1**).

*The authors thank our current lab members for thoughtful discussion on the manuscript.*

*This work was supported by a grant of the Korea Dementia Research Project through the Korea Dementia Research Center (RS-2022-KH126506 to JSL), the ABC-based Regenerative BioTherapeutics (ABC project) grant (RS-2024-00426031 to JSL), and NRF Grant (2022R1A2C3013138 to HR, RS-2024-00449723 to JGL) funded by the Korea government (the Ministry of Health & Welfare,* *Ministry of Science and ICT, and Ministry of Education).*
